# Clinical impact of atrial fibrillation progression in patients with heart failure with preserved ejection fraction: A report from the CHART-2 Study

**DOI:** 10.1093/europace/euae218

**Published:** 2024-08-16

**Authors:** Tomohiro Ito, Takashi Noda, Kotaro Nochioka, Takashi Shiroto, Nobuhiko Yamamoto, Hiroyuki Sato, Takahiko Chiba, Yuhi Hasebe, Makoto Nakano, Hiroyuki Takahama, Jun Takahashi, Satoshi Miyata, Hiroaki Shimokawa, Satoshi Yasuda

**Affiliations:** Department of Cardiovascular Medicine, Tohoku University Graduate School of Medicine, Sendai, Japan; Department of Cardiovascular Medicine, Tohoku University Graduate School of Medicine, Sendai, Japan; Department of Cardiovascular Medicine, Tohoku University Graduate School of Medicine, Sendai, Japan; Department of Cardiovascular Medicine, Tohoku University Graduate School of Medicine, Sendai, Japan; Department of Cardiovascular Medicine, Tohoku University Graduate School of Medicine, Sendai, Japan; Department of Cardiovascular Medicine, Tohoku University Graduate School of Medicine, Sendai, Japan; Department of Cardiovascular Medicine, Tohoku University Graduate School of Medicine, Sendai, Japan; Department of Cardiovascular Medicine, Tohoku University Graduate School of Medicine, Sendai, Japan; Department of Cardiovascular Medicine, Tohoku University Graduate School of Medicine, Sendai, Japan; Department of Cardiovascular Medicine, Tohoku University Graduate School of Medicine, Sendai, Japan; Department of Cardiovascular Medicine, Tohoku University Graduate School of Medicine, Sendai, Japan; Teikyo University Graduate School of Public Health, Tokyo, Japan; Department of Cardiovascular Medicine, Tohoku University Graduate School of Medicine, Sendai, Japan; International University of Health and Welfare, Narita, Japan; Department of Cardiovascular Medicine, Tohoku University Graduate School of Medicine, Sendai, Japan

**Keywords:** Heart failure with preserved ejection fraction, Atrial fibrillation, Progression, Prognosis

## Abstract

**Aims:**

Atrial fibrillation (AF) frequently coexists with heart failure with preserved ejection fraction (HFpEF), and clinical outcomes of patients with AF vary depending on its subtype. While AF progression characterized by the transition from paroxysmal AF to persistent AF is sometimes observed, the incidence and clinical impact of AF progression in patients with HFpEF remain to be explored.

**Methods and results:**

We enrolled patients with HFpEF and paroxysmal AF from the Chronic Heart Failure Analysis and Registry in the Tohoku District-2 (CHART-2) Study. AF progression was defined as the transition from paroxysmal AF to persistent AF. A total of 718 patients (median age: 72 years, 36% were female) were enrolled. For a median follow-up of 6.0 years (interquartile range: 3.0–10.2 years), AF progression occurred in 105 patients (14.6%), with a cumulative incidence of 16.7% at 10 years. In the multivariable Cox proportional hazards model, previous hospitalization for heart failure [hazard ratio (HR) 1.74, 95% confidence interval (CI) 1.16–2.60; *P* = 0.007] and left atrial diameter (per 5-mm increase) (HR 1.37, 95% CI 1.20–1.55; *P* < 0.001) were significantly associated with AF progression. Furthermore, AF progression was significantly linked to worsening heart failure (adjusted HR 1.68, 95% CI 1.18–2.40; *P* = 0.004). Notably, 27 cases (26%) of worsening heart failure occurred within 1 year following AF progression.

**Conclusion:**

In patients with HFpEF, AF progression is significantly associated with adverse outcomes, particularly worsening heart failure. An increased risk is observed in the early phases following progression to persistent AF.

**Registration:**

Clinical Trials.gov Identifier: NCT00418041

## Graphical Abstract

**Graphical abstract:**
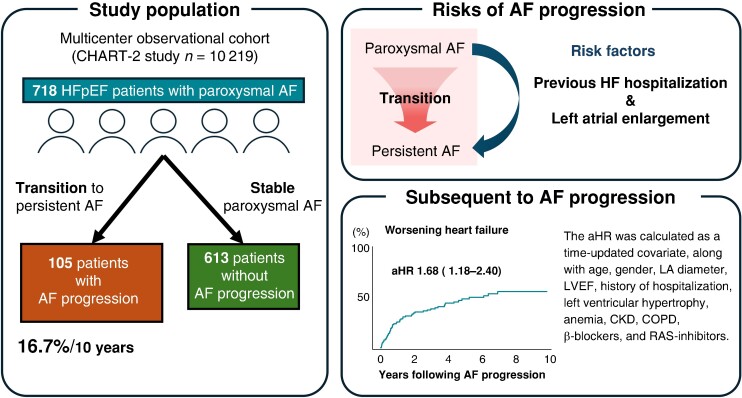

## Introduction

Atrial fibrillation (AF) stands as the most prevalent cardiac arrhythmia globally.^1^ Its prevalence mirrors that of heart failure, affecting millions of adults worldwide.^2^ AF not only predisposes individuals to the development of heart failure, but also increases the risk of adverse outcomes.^2–4^ Nearly half of heart failure cases fall under heart failure with preserved ejection fraction (HFpEF), where AF prevails, accounting for 45.2 to 65% of cases, and is linked to heightened cardiovascular risk.^5,6^ A recent study highlighted that those adverse outcomes, especially those associated with worsening heart failure, were more prevalent among HFpEF patients with AF.^7^ The progression of AF defined as the shift from paroxysmal AF to persistent AF confirmed by electrocardiogram (ECG) is a common occurrence in clinical settings, with reported rates ranging from 27 to 36% over 10 years.^8,9^ This progression is associated with serious adverse events, such as stroke, systemic embolism, hospitalization for heart failure, and other cardiovascular morbidities and mortalities.^9–11^ However, many aspects of the interaction between HFpEF and AF remain unclear, particularly concerning the clinical implications of AF progression in HFpEF patients. In this study, we aimed to address this knowledge gap by investigating the incidence of AF progression, identifying predictive risk factors for such progression, and examining the prognosis following AF progression among HFpEF patients in our registry study, the Chronic Heart Failure Analysis and Registry in the Tohoku District-2 (CHART-2) Study.^12^ Additionally, we sought to evaluate the annual event rate following AF progression to better understand its impact on overall prognosis.

## Methods

### Study design

The CHART-2 Study is a multicenter, prospective observational cohort study.^12^ Briefly, the CHART-2 Study enrolled 10 219 consecutive patients aged 20 years or older who presented with either coronary artery disease (Stage A HF by the ACC/AHA guidelines, n = 868), structural heart disease without symptoms (Stage B HF, n = 4514), or a current or past history of symptomatic heart failure (Stage C/D HF, n = 4837) from 24 affiliated hospitals between October 2006 and March 2010. Detailed patient information, including medical history, laboratory data, ECG data, and echocardiography data, were collected at the time of enrolment. Clinical data were reviewed annually by clinical research coordinators through medical records, surveys, and telephone interviews. The present study adhered to the principles of the Declaration of Helsinki and was registered on ClinicalTrials.gov (identifier NCT00418041). Institutional review board approval was obtained from each field centre, and written informed consent was obtained from all patients.

**Figure 1:**
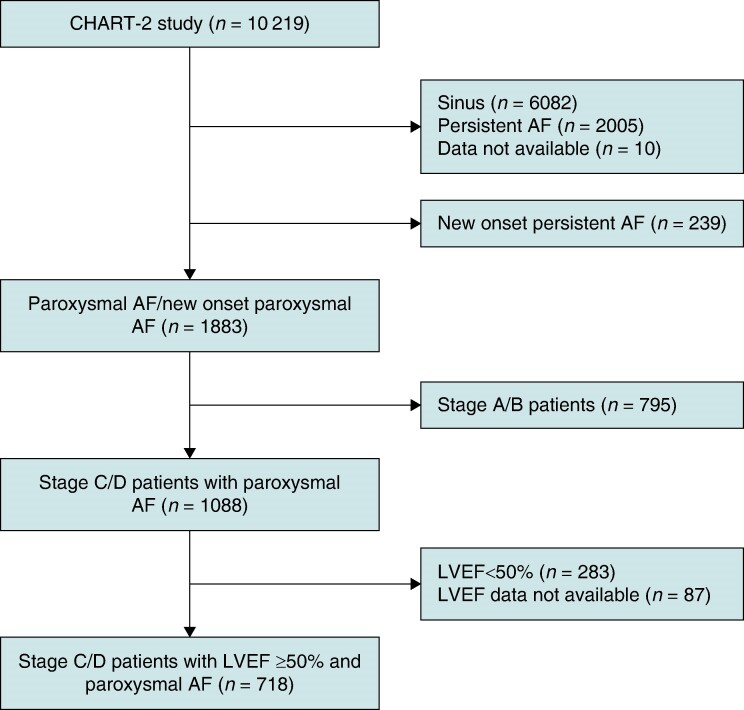
**Study flowchart.** A total of 718 patients with stage C/D heart failure and paroxysmal AF were included in the analysis. AF, atrial fibrillation; LVEF, left ventricular ejection fraction.

Out of the 10 219 patients enrolled in the CHART-2 Study, 1883 had either a history of paroxysmal AF or experienced new-onset paroxysmal AF during the observation period. From this subset of patients, we initially selected 1088 consecutive patients diagnosed with Stage C/D HF. Patients without left ventricular ejection fraction (LVEF) data were excluded from the analysis. Specifically, we included 718 HF patients with an LVEF of 50% or higher at the time of paroxysmal AF diagnosis, consistent with the universal definition of HFpEF.^13^  *Figure 1* outlines the study flow leading to the final enrolment of patients with paroxysmal AF and Stage C/D HFpEF.

### Definition and outcomes

AF was diagnosed as either paroxysmal AF or persistent AF based on its clinical characteristics and duration according to the Japanese guidelines established in 2008 at the time of enrolment.^14^ Paroxysmal AF was defined as AF lasting within 7 days, while persistent AF was defined as AF continuing for over 7 days. The same data collected at registration were reclassified annually based on repeated medical record reviews during the study period. Since 2013, the reclassification has followed the 2013 guidelines.^15^ Paroxysmal AF was not classified in detail due to insufficient data on electrocardioversion or catheter ablation, particularly regarding whether patients had previously experienced persistent AF or had received interventional treatment. The 12-lead ECG data were recorded during patients’ regular visits to the participating hospital. Additionally, ECGs were recorded when patients experienced symptoms or as deemed necessary by the attending physician. The transition of AF from paroxysmal to persistent type was determined by certified cardiologists at each institute based on an assessment of ECG data and clinical course. The event was further evaluated by an independent data monitoring committee.

The primary endpoint of this study was worsening heart failure, which included hospitalization for HF and the development of symptomatic HF in outpatient settings, as defined by the Framingham Heart Failure Criteria.^16^ In cases where events occurred multiple times during the follow-up period, only the first event was considered. Additionally, we analysed baseline characteristics to identify risk factors for AF progression in patients with paroxysmal AF. In a secondary analysis, all patients were divided into two groups: those with and those without AF progression. Clinical outcomes following AF progression were then examined. To address time-to-event bias, landmark analysis was performed. Specifically, at 1, 4, and 7 years after enrolment, patients were categorized into groups with and without AF progression, and the incidence of worsening HF was compared. Only patients who survived at each respective time point were included in the analysis.

### Statistical analysis

Continuous variables were presented as mean ± standard deviation or as median with interquartile range (IQR), while categorical data were expressed as frequency (percentage). Comparisons of these variables were conducted using Welch's *t*-test for continuous variables with normal distribution, Fisher's exact test for categorical variables, and the Mann–Whitney *U* test for continuous variables with non-normal distribution.

In the primary analysis, we estimated the incidence rate of AF progression considering all-cause mortality as a competing risk. Univariable and multivariable analyses were performed using the Fine–Gray sub-distribution hazard model to identify independent risk factors for AF progression. Risk factors with a significance level of *P* < 0.10 in univariable analysis and predefined covariates based on previous studies were included in the multivariable analysis.^10,17,18,19,20^ These covariates were age (≥75 years), gender, body mass index (per 1-kg/m² increase), hypertension, diabetes mellitus, previous hospitalization for HF, previous stroke/transient ischemic attack, previous myocardial infarction, left atrial (LA) diameter (per 5-mm increase), left ventricular hypertrophy, and the use of medications. For the secondary analysis, we developed a cumulative incidence curve of worsening HF following AF progression. Subsequently, we constructed a Cox proportional hazards regression model to assess the relative hazard of worsening HF before or after AF progression, treating it as a time-updated covariate. Covariates were predefined as age (≥75 years), gender, anaemia, chronic kidney disease, chronic obstructive pulmonary disease, previous hospitalization for HF, LA diameter (≥45 mm), LVEF (<60%), left ventricular hypertrophy, and the use of medications. Additionally, to compare outcomes between patients with and without AF progression, we conducted a Fine–Gray sub-distribution hazard model in the landmark analysis. Covariates in patients with AF progression were collected at the time of AF progression. In each model, all-cause mortality was considered as a competing risk.

All statistical analyses were conducted using R software (version 4.3.2).^21^ A two-sided *P*-value < 0.05 was considered to be statistically significant for all analyses.

## Results

### Incidence of AF progression

**Figure 2:**
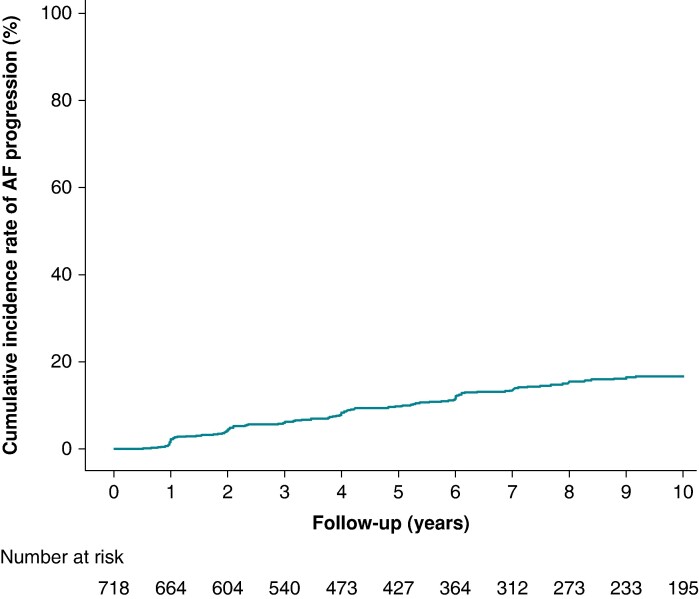
**Cumulative incidence rate of AF progression.** The cumulative incidence rate of AF progression was calculated for 718 patients. Incidence rates were calculated with all-cause mortality as a competing risk.

Finally, we enrolled 718 patients with paroxysmal AF and Stage C/D HFpEF. *Table 1* shows the baseline characteristics of all study patients, as well as those stratified by AF progression. The median age of the cohort was 72 years [interquartile range (IQR): 65–79], with 261 patients (36%) being female. Over a median follow-up period of 6.0 years (IQR: 3.0–10.2 years), AF progression was noted in 105 patients (14.6%). The cumulative incidence of AF progression considering all-cause mortality as a competing risk was 9.7% at 5 years and 16.7% at 10 years (*Figure 2*). The duration from the last confirmation of paroxysmal AF to the diagnosis of persistent AF was a median of 0.99 years (IQR 0.93–1.1, minimum 0.49, maximum 1.9). During the observation period, we observed a gradual decline in LVEF while maintaining LVEF ≥ 50%. However, we did not observe any significant change before and after AF progression (see Supplementary material online, *Figure S1*).

**Table 1:** Patient baseline characteristics

	All (n = 718)	(−) AF progression (n = 613)	(+) AF progression (n = 105)	*P*-value
Age (years)	72 ± 11	72 ± 11	71 ± 10	0.332
Gender (female)	261 (36)	218 (36)	43 (41)	0.323
BMI (kg/m^2^)	23.8 ± 3.8	23.9 ± 3.8	23.8 ± 3.8	0.946
Systolic BP (mmHg)	128 ± 20	128 ± 19	126 ± 21	0.314
Diastolic BP (mmHg)	71 ± 12	71 ± 12	70 ± 12	0.266
Heart rate (/min)	69 ± 16	70 ± 16	68 ± 15	0.389
**NYHA class**				
Ⅰ	180 (25)	159 (26)	21 (20)	0.453
Ⅱ	487 (68)	412 (68)	75 (71)	
Ⅲ	46 (6)	37 (6)	9 (9)	
Ⅳ	2 (0.3)	2 (0.3)	0 (0)	
**Risk factors**				
Hypertension	664 (92)	565 (92)	99 (94)	0.551
Diabetes mellitus	261 (36)	229 (37)	32 (30)	0.189
Dyslipidaemia	606 (84)	520 (85)	86 (82)	0.467
Smoking	306 (43)	267 (44)	39 (37)	0.236
Alcohol	332 (46)	284 (46)	48 (46)	0.982
Past drinking	70 (10)	61 (10)	9 (9)	
Occasional drinking	92 (13)	78 (13)	14 (13)	
Daily drinking	170 (24)	145 (24)	25 (24)	
CHADS2 score	3 (2–4)	3 (2–4)	3 (2–4)	0.515
**Previous history**				
Stroke	142 (20)	126 (21)	16 (15)	0.234
Malignant disease	132 (18)	114 (19)	18 (17)	0.786
COPD	40 (6)	32 (5)	8 (8)	0.354
Hospitalization for HF	335 (47)	271 (44)	64 (61)	0.002
Myocardial infraction	177 (25)	171 (28)	17 (16)	0.012
HCM	44 (6.1)	36 (6)	8 (8)	0.508
**Echocardiogram**				
LVDd (mm)	49 ± 7	49 ± 7	48 ± 7	0.325
LVDs (mm)	31 ± 6	31 ± 6	30 ± 6	0.299
LA diameter (mm)	42 ± 7	42 ± 7	45 ± 7	<0.001
LVEF (%)	66 ± 9	66 ± 9	66 ± 8	0.509
LVH	425 (59)	360 (59)	65 (62)	0.668
MR	62 (9)	54 (9)	8 (8)	0.851
TRPG	28 ± 14	28 ± 15	27 ± 10	0.586
**Laboratory findings**				
Hemoglobin (g/dL)	13.0 ± 1.9	13.0 ± 1.9	13.3 ± 1.9	0.157
Anemia	263 (37)	231 (38)	32 (31)	0.188
eGFR (mL/min/1.73 m^2^)	59 (46–72)	59 (46–72)	59 (46–73)	0.856
CKD	365 (51)	309 (51)	56 (53)	0.673
Albumin (mg/dL)	4.1 ± 0.4	4.1 ± 0.4	4.1 ± 0.4	0.598
LDL-cho (mg/dL)	103 ± 30	103 ± 30	102 ± 32	0.669
HbA1c (%)	6.2 ± 0.9	6.2 ± 0.9	6.2 ± 0.9	0.969
BNP (pg/mL)	103 (47–216)	100 (46–207)	120 (72–236)	0.075
**Medication**				
β-Blockers	332 (46)	276 (45)	56 (53)	0.138
RAS-inhibitors	481 (67)	405 (66)	76 (72)	0.218
MRA	133 (19)	112 (18)	21 (20)	0.684
Diuretics	330 (46)	281 (46)	49 (47)	0.916
Furosemide dose	20 (20–40)	20 (20–40)	20 (20–40)	0.873
Statins	269 (37)	228 (37)	31 (30)	0.153
Antiplatelet	395 (55)	344 (56)	51 (49)	0.168
Anticoagulant	295 (41)	239 (39)	56 (53)	0.007
Anti-arrhythmic drugs	172 (24)	144 (24)	28 (27)	0.536

Variables are presented as mean and SD or median and interquartile range or total numbers and percentages. AF, atrial fibrillation; BMI, body mass index; BNP, B-type natriuretic peptide; BP, blood pressure; CHADS2, congestive heart failure (1 point), hypertension (1 point), age ≥75 years (1 point), diabetes mellitus (1 point), prior stroke or TIA or thromboembolism (2 points); CKD, chronic kidney disease (eGFR <60 mL/min/1.73 m^2^); COPD, chronic obstructive pulmonary disease; HCM, hypertrophic cardio myopathy; HF, heart failure; LA, left atrial; LVDd, left ventricular end diastolic diameter; LVDs, left ventricular end systolic diameter; LVEF, left ventricular ejection fraction; LVH, left ventricular hypertrophy; MR, mitral regurgitation including moderate MR or severe MR. MRA, mineralocorticoid receptor antagonist; NYHA, New York Heart Association; RAS, renin-angiotensin system; TRPG, tricuspid regurgitation peak gradient.

### Identifying risk factors for AF progression

Patients were divided into two groups based on the AF progression status (*Table 1*). Patients with AF progression had a higher incidence of prior hospitalization for HF (61 vs. 44%), a lower incidence of prior myocardial infarction (16 vs. 28%), a larger LA diameter (45 ± 7 mm vs. 42 ± 7 mm), and a higher rate of anticoagulant therapy usage (53 vs. 39%). Age did not emerge as an independent risk factor possibly due to the U-shaped relationship observed between age and AF progression in this cohort (see Supplementary material online, *Figure S2*). In the multivariable Cox proportional hazard model, which included factors identified through univariable analysis (*Table 2*), the following variables were significantly associated with AF progression; previous hospitalization for HF [hazard ratio (HR) 1.74, 95% confidence interval (CI) 1.16–2.60; *P* = 0.007] and LA diameter (HR 1.37, 95% CI 1.20–1.55; *P* < 0.001) per 5-mm increase. These associations remained unchanged, even after adjusting for the use of β-blockers, RAS inhibitors, or antiarrhythmic drugs in Model 2. For a sensitivity analysis, we performed another multivariable analysis treating age and LA diameter as continuous variables: age (per 5-year increase) and LA diameter (per 1-mm increase), respectively (see Supplementary material online, *Table S1*).

**Table 2:** Multivariable predictors for AF progression

			Multivariable analysis			
	Univariable analysis		Model 1		Model 2	
	HR (95% CI)	*P*-value	HR (95% CI)	*P*-value	HR (95% CI)	*P*-value
Age (≥75 years)	1.46 (0.99–2.15)	0.06	1.48 (0.99–2.23)	0.06	1.47 (0.98–2.21)	0.16
Gender (female)	1.20 (0.81–1.77)	0.37	1.13 (0.76–1.60)	0.55	1.19 (0.79–1.78)	0.40
Body mass index	0.98 (0.93–1.04)	0.52	0.96 (0.91–1.02)	0.19	0.96 (0.90–1.01)	0.12
Hypertension	1.59 (0.67–3.64)	0.27	1.83 (0.79–4.24)	0.16	1.69 (0.72–3.98)	0.23
Diabetes mellitus	0.80 (0.53–1.22)	0.30	0.85 (0.54–1.41)	0.44	0.85 (0.55–1.32)	0.47
Previous hospitalization for HF	1.94 (1.31–2.87)	<0.001	1.74 (1.16–2.60)	0.007	1.66 (1.10–2.49)	0.015
Previous stroke	0.81 (0.48–1.38)	0.44	0.88 (0.51–1.51)	0.64	0.90 (0.52–1.54)	0.69
Previous myocardial infraction	0.55 (0.33–0.93)	0.03	0.60 (0.35–1.04)	0.07	0.58 (0.33–1.01)	0.054
LA diameter	1.37 (1.21–1.55)	<0.001	1.37 (1.20–1.55)	<0.001	1.37 (1.21–1.56)	<0.001
Left ventricular hypertrophy	1.15 (0.77–1.70)	0.49	0.98 (0.65–1.47)	0.91	0.92 (0.61–1.39)	0.70
β-Blockers	1.16 (0.70–1.93)	0.56			1.23 (0.82–1.85)	0.31
RAS-inhibitors	1.34 (0.87–2.06)	0.18			1.30 (0.82–2.06)	0.26
Anti-arrhythmic drugs	0.98 (0.64–1.52)	0.94			0.92 (0.59–1.43)	0.71

A maximum of 13 (1.8%) observations were deleted because of missing variables. LA diameter per 5-mm increase. AF, atrial fibrillation; HF, heart failure; LA, left atrial; RAS, renin–angiotensin system.

### Worsening heart failure following AF progression

**Figure 3:**
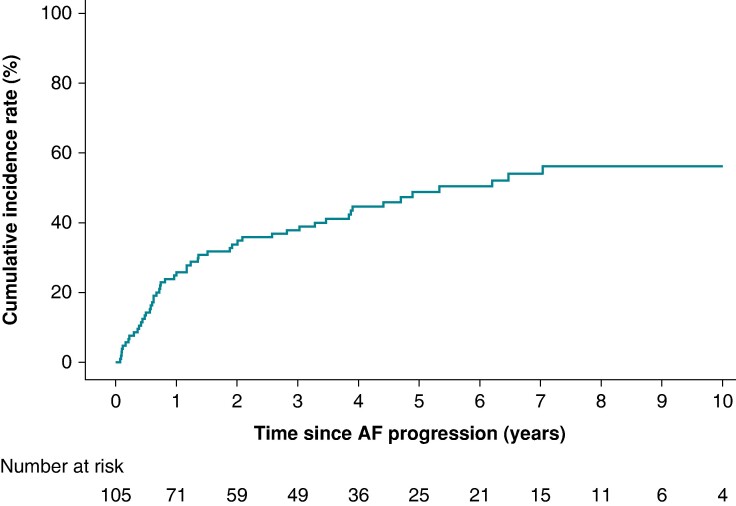
**Cumulative incidence rate of worsening heart failure subsequent to AF progression.** Incidence rates were calculated with all-cause mortality as a competing risk.

We compared the clinical outcomes between patients with and those without AF progression. Baseline characteristics at the time of AF progression were described for patients with AF progression (n = 105) in Supp`lementary material online, *Table S2*. Among patients experiencing AF progression, worsening HF was observed in 52 individuals (49.5%) during a median follow-up period of 2.8 years (IQR 0.7–4.8) (*Figure 3*). The estimated event rate of worsening HF was 48.8% at 5 years and 56.1% at 10 years.

**Figure 4:**
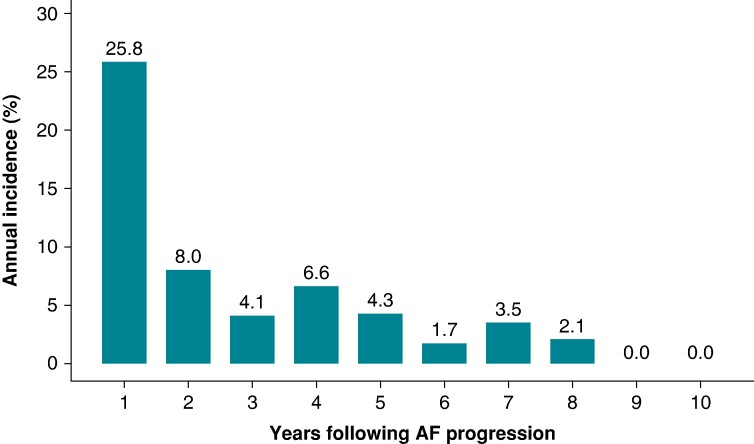
**Landmark analysis of cumulative incidence rate of worsening heart failure every 3** **years.** The landmark analysis depicts the incidence of worsening heart failure at each 3-year observation period. Patients with AF progression showed a significantly higher incidence of worsening heart failure compared to those without AF progression. Hazard ratios for AF progression adjusted for age (≥75 years) and sex remained consistent across the observation periods. Hazard ratios were calculated with all-cause mortality as a competing risk.

Landmark analysis revealed a consistent trend in the incidence of worsening HF in both groups at each 3-year observation period (*Figure 4*). After adjustment for age (≥75 years) and sex, AF progression was associated with an increased risk of worsening HF, with adjusted hazard ratios (aHRs) of 2.68 (CI 1.13–6.35) in the first observation period, 1.42 (CI 0.80–2.54) in the second period, and 1.79 (CI 1.03–3.11) in the last period (*Figure 4*).

### Clinical predictors of worsening heart failure

In the Cox proportional hazards regression model utilizing AF progression as a time-updated covariate, AF progression demonstrated a significant association with an increased risk of worsening HF [adjusted HR (aHR) 1.68, 95% CI 1.18–2.40; *P* = 0.004], independent of covariates previously identified in HFpEF patients (*Table 3*). Following stepwise variable selection based on the AIC, AF progression maintained its status as an independent risk factor for worsening HF (aHR 1.71, 95% CI 1.20–2.42; *P* = 0.003), alongside variables, such as age (≥75 years), chronic renal failure, history of hospitalization for HF, and left ventricular hypertrophy. For a sensitivity analysis, we performed another multivariable analysis treating age, LA diameter, and LVEF as continuous variables: age (per 5-year increase), LA diameter (per 1-mm increase), and LVEF (per 1% increase), respectively (see Supplementary material online, *Table S3*). This pattern persisted in analyses where the primary outcome was replaced by a composite event of worsening heart failure and all-cause mortality.

**Table 3:** Cox proportional hazards regression model for worsening heart failure

	Multivariable analysis			
	Model 1		Model 2	
	HR (95% CI)	*P*-value	HR (95% CI)	*P*-value
AF progression	1.68 (1.18–2.40)	0.004	1.71 (1.20–2.43)	0.003
Age (≥75 years)	1.07 (0.83–1.36)	0.61		
Gender (female)	1.99 (1.55–2.56)	<0.001	1.92 (1.51–2.43)	<0.001
Anaemia	1.51 (1.20–1.91)	0.001	1.51 (1.20–1.91)	<0.001
Chronic kidney disease	1.33 (1.04–1.69)	0.021	1.33 (1.04–1.68)	0.021
COPD	0.74 (0.47–1.16)	0.19		
Previous hospitalization for HF	1.71 (1.36–2.15)	<0.001	1.73 (1.39–2.17)	<0.001
LA diameter (>45 mm)	1.32 (1.04–1.67)	0.020	1.31 (1.04–1.66)	0.023
LVEF (<60%)	1.20 (0.93–1.54)	0.16		
Left ventricular hypertrophy	1.31 (1.03–1.66)	0.030	1.35 (1.07–1.70)	0.013
β-Blockers	0.75 (0.59–0.95)	0.016	0.77 (0.61–0.98)	0.030
RAS-inhibitors	1.13 (0.90–1.43)	0.30		

A maximum of 16 (2.2%) observations were deleted because of missing variables. AF progression was considered as a time-updated covariate. In Model 2, covariables were extracted with step-wise selection based on AIC. AF, atrial fibrillation; COPD, chronic obstructive pulmonary disease; HF, heart failure; LA, left atrial; LVEF, left ventricular ejection fraction; RAS, renin–angiotensin system.

### Incidence of worsening heart failure post following AF progression

**Figure 5:**
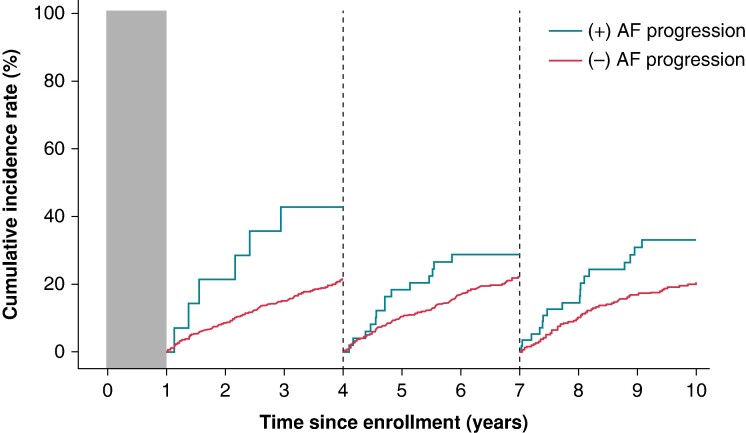
**Annual incidence rate of worsening heart failure following AF progression.** The annual incidence of worsening heart failure was most frequent in the first year (25.8% per year), followed by a subsequent decrease in the second year (8.8% per year). These rates were calculated with all-cause mortality considered as a competing risk.

By focusing on 105 patients with AF progression, we observed the annual incidence of worsening HF most frequently in the first year (25.8% per year), followed by the second year (8.8% per year) (*Figure 5*). Thereafter, a gradual downward trend was observed, with about half of all patients experiencing worsening HF within 5 years (*Figure 5*).

## Discussion

In this study, we found a significant association between AF progression and adverse outcomes related to worsening HF. Moreover, we observed that the risk of these outcomes was increased within the first few years following progression to persistent AF in HFpEF patients.

### Incidence of AF progression

In our study, we observed an AF progression incidence rate of 16.7% over a 10-year period among HFpEF patients, which demonstrated a consistent trend throughout the observation period. This rate appears to be lower compared to the previous studies involving both HF and non-HF populations, where AF progression rates ranged from 27 to 36% over 10 years, approximately 5 to 6% per year.^8,9^

One potential explanation for the lower incidence rate of AF progression observed in our study could be attributed to the strict control of comorbidities, particularly hypertension, among our study subjects. Notably, our patients exhibited well-controlled blood pressure, with over half maintaining levels below 130 mmHg. The stringent management of comorbid conditions, such as hypertension, in patients with HFpEF and paroxysmal AF might have contributed to the observed reduction in AF progression. However, further study is warranted to elucidate the precise mechanisms underlying the observed differences in AF progression rates and to validate the potential impact of comorbidity control on AF progression in HFpEF patients.

### Factors associated with AF progression

The present study highlighted that previous hospitalization for HF and LA enlargement was significantly associated with an increased risk of AF progression in HFpEF patients. A recent meta-analysis encompassing patients with and without HF identified several clinical risk factors linked to AF progression, including age, hypertension, obesity, LA enlargement, history of HF, and prolonged duration of paroxysmal AF.^18^

Additionally, a multicenter cohort study developed the HATCH score for predicting AF progression, which includes hypertension, age ≥75 years, previous transient ischemic attack or stroke, chronic obstructive pulmonary disease, and HF.^19^ While there is limited literature specifically addressing risk factors for AF progression in HFpEF patients, the present findings align with previous reports involving patients without HFpEF. Notably, although hypertension, age, LA enlargement, and history of HF were commonly implicated across several studies, hypertension did not emerge as an independent risk factor in our analysis likely due to its high prevalence (over 90%) among our patient cohort. In the present study, age was not identified as an independent risk factor due to its U-shaped relationship with AF progression. When dividing the entire study population into three groups, aged under 65, aged between 65 and 75, and aged over 75, the prevalence of previous hospitalization for HF in each group was 51.9, 41.2, and 47.8%, respectively, while the mean atrial diameter was observed to be 41.5, 41.6, and 42.9 mm, respectively. These disparities in the prevalence of previous hospitalization for HF likely explain the U-shaped relationship between age and AF progression. In younger patients, a higher prevalence of previous hospitalization for HF was observed. Therefore, we speculate that the severity of heart failure indicated by a higher history of past hospitalization may have impacted AF progression in patients under 65 years old.

Furthermore, LA enlargement emerged as a pivotal factor for AF progression, consistent with the current understanding of AF pathophysiology. Atrial remodeling driven by increased filling pressure and atrial overload can lead to atrial remodeling characterized by structural changes in the atria, ultimately fostering conditions conducive to the perpetuation of AF.^22,23^

### Adverse effects of AF progression and their temporal trends

AF progression was found to be significantly associated with an increased risk of worsening heart failure in our study, even after adjusting for several factors. Consistent with our findings, previous studies have reported that AF progression is linked to adverse events, such as thromboembolism, heart failure exacerbations, cardiovascular events, and all-cause mortality.^11,19^ For subclinical AF, an increase in atrial high-rate episodes detected by cardiac implantable devices was associated with an increased risk of hospitalization for HF.^24^

While definitions of AF progression may vary across studies, the detrimental effects of AF progression appear to be consistent. For example, Ogawa et al. demonstrated an increased incidence of stroke associated with AF progression.^10^ Interestingly, in our study, we did not observe an increase in stroke or myocardial infarction following AF progression despite the low use of anticoagulants. However, this finding may be influenced by the limited number of observed events.

Furthermore, we observed that the clinical adverse effects of AF progression primarily occurred within a year after its onset. Similarly, in the new occurrence of AF in symptomatic HF patients, cardiovascular events were noted, especially shortly after its onset as well.^3,25^ This temporal pattern aligns with the physiological consequences of AF progression, wherein the loss of atrial contraction and subsequent atrioventricular desynchronization can lead to worsening haemodynamics. Specifically, compared to sinus rhythm, AF is characterized by irregular heart rates, which can negatively impact cardiac output, and the absence of atrial contraction, which impairs left ventricular filling and ventricular stroke volume.^26,27^ Such alteration in cardiac rhythm considered as progression of atrial cardiomyopathy may play a crucial role in HF patients; particularly, patients with HFpEF are susceptible to decompensation in the early phases following AF progression.^28^

### Clinical implication: potential interventions for AF prevention and management in HFpEF

Given the observed increase in adverse events early after AF progression, it is imperative to prioritize interventions before progression occurs, specifically during the paroxysmal AF stage. Interventions for paroxysmal AF typically involve modifying risk factors, while medical therapy and catheter ablation are considered for rhythm control. However, it is noteworthy that many antiarrhythmic drugs used for HF patients have been associated with poor prognosis, particularly considering the common coexistence of chronic renal failure with HFpEF, which renders antiarrhythmic drugs less favourable in this context.^29^

Although there is no complete validation for catheter ablation in HFpEF patients due to the absence of large randomized controlled trials targeting this population, some potential benefits have recently emerged. For instance, in an observational study, Xie et al. reported that catheter ablation in HFpEF patients resulted in a reduction in rehospitalization for worsening heart failure and AF recurrence.^30^ Randomized controlled trials have demonstrated improvements in haemodynamics after catheter ablation.^31^ Additionally, a post-hoc analysis of the CABANA trial suggests potential benefits for HFpEF patients with NYHA ≥2, although further study is needed to confirm the absolute effectiveness of catheter ablation in HFpEF.^32^ Recent trials, such as EARLY-AF and STOP-AF, have highlighted the role of catheter ablation as an initial treatment for patients with paroxysmal AF.^33,34^ The EARLY-AF trial also suggests that early intervention may reduce progression to persistent AF.^33^ Implementing interventions for paroxysmal AF before AF progression may therefore lead to improved clinical outcomes in patients with HFpEF, thereby mitigating adverse outcomes associated with AF progression. Further studies aimed at elucidating the optimal timing and selection of interventions for AF management in HFpEF patients are crucial for advancing clinical practice and improving patient outcomes.

### Study limitations

Several limitations should be mentioned for the present study. First, the inability to assess the burden of paroxysmal AF due to the lack of continuous ECG monitoring represents a significant limitation. Consequently, the classification of patients was based solely on ECG data collected during routine clinical practice, potentially leading to underestimation or misclassification of AF progression. Additionally, patients with a previous hospitalization for HF may have had closer follow-up, increasing their chances of receiving an ECG. Second, the availability of echocardiographic data on LV diastolic function was limited, which hindered a comprehensive assessment of the detailed status of HFpEF. We did not have data on past LVEF measurements, so we could not differentiate patients who may have had a previous LVEF <50%. Third, we noted a relatively low rate of anticoagulation use, which aligns with the 40–60% of patients reported in previous studies conducted during a similar period to the CHART-2 Study.^35,36,37^ This may be related, at least in part, with the fact that the evidence was not as robust when the CHART-2 Study enrolled patients and concerns about bleeding risk, particularly among elderly patients (e.g. those over 80 years old) or those with cancer. Fourth, the scarcity of data on catheter ablation prevented a thorough examination of its impact on the study population, and the same applies to electrical cardioversion. It is therefore difficult to evaluate whether restoration of sinus rhythm was targeted. Fifth, the lack of information on the amount of alcohol consumed constrained our analysis of risk factors for AF progression. Finally, caution is warranted when generalizing the results to other populations as the CHART-2 Study is an observational study conducted in Japan.

## Conclusion

In conclusion, our study highlights a significant association between AF progression and an increased risk of worsening HF in patients with HFpEF. Notably, this risk appears to be most pronounced within the first year following AF progression.

## Supplementary Material

euae218_Supplementary_Data

## Data Availability

The CHART-2 Study data are available upon reasonable request to the corresponding author. This study was approved by the Ethics Committee of Tohoku University Graduate School of Medicine (Reference no. 2021-1-634).
