# Supplementary material for: Clinical impact of atrial fibrillation progression in patients with heart failure with preserved ejection fraction: A report from the CHART-2 Study
Source: Europace. 2024 Aug 16;26(9):euae218. doi: 10.1093/europace/euae218 (PMC11368130; doi:10.1093/europace/euae218)
Supplement: euae218_Supplementary_Data [file euae218_supplementary_data.zip › R3 Supplementary table.docx]

**Supplementary Table 1.** **Multivariable predictors for AF progression with age, BMI, and LA diameter treated as continuous variables.**

|  | Univariable analysis | |  | Multivariable analysis | | | | | |
| --- | --- | --- | --- | --- | --- | --- | --- | --- | --- |
|  |  | |  | Model 1 | | |  | Model 2 | |
|  | HR (95% CI) | P-value |  | HR (95% CI) | | P-value |  | HR (95% CI) | P-value |
| Age | 1.07 (0.98-1.18) | 0.13 |  | 1.08 (0.98-1.19) | | 0.14 |  | 1.09 (0.98-1.20) | 0.10 |
| Gender (female) | 1.20 (0.81-1.77) | 0.37 |  | 1.15 (0.77-1.71) | | 0.50 |  | 1.21 (0.81-1.80) | 0.36 |
| Body mass index | 0.98 (0.93-1.04) | 0.52 |  | 0.96 (0.91-1.02) | | 0.20 |  | 0.96 (0.90-1.01) | 0.12 |
| Hypertension | 1.59 (0.67-3.64) | 0.27 |  | 1.83 (0.79-4.26) | | 0.16 |  | 1.67 (0.71-3.95) | 0.24 |
| Diabetes mellitus | 0.80 (0.53-1.22) | 0.30 |  | 0.85 (0.55-1.32) | | 0.47 |  | 0.85 (0.55-1.33) | 0.48 |
| Previous hospitalization for HF | 1.94 (1.31-2.87) | <0.001 |  | 1.76 (1.18-2.64) | | 0.006 |  | 1.68 (1.12-2.52) | 0.01 |
| Previous stroke | 0.81 (0.48-1.38) | 0.44 |  | 0.89 (0.52-1.54) | | 0.68 |  | 0.91 (0.53-1.57) | 0.74 |
| Previous myocardial infraction | 0.55 (0.33-0.93) | 0.03 |  | 0.63 (0.37-1.10) | | 0.11 |  | 0.60 (0.34-1.05) | 0.07 |
| LA diameter | 1.07 (1.04-1.09) | <0.001 |  | 1.07 (1.04-1.10) | | <0.001 |  | 1.07 (1.04-1.10) | <0.001 |
| Left ventricular hypertrophy | 1.15 (0.77-1.70) | 0.49 |  | 0.96 (0.64-1.44) | | 0.84 |  | 0.90 (0.59-1.36) | 0.61 |
| β-blockers | 1.16 (0.70-1.93) | 0.56 |  |  |  |  |  | 1.28 (0.85-1.93) | 0.24 |
| RAS-inhibitors | 1.34 (0.87-2.06) | 0.18 |  |  |  |  |  | 1.32 (0.83-2.08) | 0.24 |
| Anti arrhythmic drugs | 0.98 (0.64-1.52) | 0.94 |  |  |  |  |  | 0.90 (0.58-1.41) | 0.66 |

A maximum of 13 (1.8%) observations were deleted because of missing variables in multivariable analysis. Age per 5-year increase; Body mass index per 1-kg/m^2^ increase; LA diameter per 1-mm increase. AF, atrial fibrillation; HF, heart failure; LA, left atrial; RAS, renin-angiotensin system.

**Supplementary Table 2. Patient characteristics at the time of AF progression**

|  | All (n=718) | (-) AF progression (n=613) | (+) AF progression (n=105) | P-value |
| --- | --- | --- | --- | --- |
| Age (years) | 71±11 | 72±11 | 74±10 | <0.001 |
| Female (%) | 261(36) | 218(36) | 43 (41) | 0.32 |
| BMI (kg/m^2^) | 23.7±3.8 | 23.9±3.8 | 23.2±3.8 | 0.14 |
| Systolic BP (mmHg) | 127±19 | 128±19 | 122±18 | <0.001 |
| Diastolic BP (mmHg) | 71±12 | 71±12 | 69±14 | 0.070 |
| Heart Rate (/min) | 71±16 | 70±16 | 76±16 | <0.001 |
| **Previous history** | | | | |
| Stroke | 150 (21) | 126 (21) | 24 (23) | 0.60 |
| Malignant disease | 137 (19) | 114 (19) | 23 (22) | 0.42 |
| COPD | 42 (6) | 32 (5) | 10 (10) | 0.11 |
| Hospitalization for HF | 341 (47) | 271 (44) | 70 (67) | <0.001 |
| Myocardial infraction | 189 (26) | 171 (28) | 18 (17) | 0.022 |
| HCM | 44 (6) | 36 (6) | 8 (8) | 0.51 |
| **Echocardiogram** | | | | |
| LVDd (mm) | 48±7 | 49±7 | 47±7 | 0.10 |
| LVDs (mm) | 31±6 | 31±6 | 31±7 | 0.92 |
| LA diameter (mm) | 42±8 | 42±7 | 47±7 | <0.001 |
| LVEF (%) | 65±9 | 66±9 | 63±10 | 0.005 |
| LVH | 423 (59) | 360 (59) | 13 (60) | 0.95 |
| MR | 67 (9) | 54 (9) | 13 (12) | 0.27 |
| TRPG | 28±14 | 28±15 | 29±11 | 0.27 |
| **Laboratory findings** | | | | |
| Hemoglobin (g/dl) | 13.0±1.9 | 13.0±1.9 | 13.0±2.0 | 0.99 |
| Anemia | 272 (38) | 231 (38) | 41 (39) | 0.83 |
| eGFR (ml/min/1.73m^2^) | 58 (44-72) | 59 (46-72) | 51 (40-64) | 0.001 |
| CKD | 378 (53) | 309 (51) | 69 (66) | 0.004 |
| Albumin (mg/dl) | 4.0±0.4 | 4.1±0.4 | 4.0±0.5 | 0.13 |
| LDL-cho (mg/dl) | 103±30 | 103±30 | 97±29 | 0.034 |
| HbA1c (%) | 6.2±0.9 | 6.2±0.9 | 6.2±0.7 | 0.77 |
| BNP (pg/ml) | 108 (52-231) | 100 (46-207) | 202 (97-310) | <0.001 |
| **Medication** | | | | |
| β-blockers | 340 (47) | 276 (45) | 64 (61) | 0.002 |
| RAS-inhibitors | 472 (66) | 405 (66) | 67 (64) | 0.739 |
| MRA | 135 (19) | 112 (18) | 23 (22) | 0.417 |
| Diuretics | 333 (46) | 281 (46) | 52 (50) | 0.53 |
| Furosemide dose | 20 (20-40) | 20 (20-40) | 20 (20-40) | 0.46 |
| Statins | 261 (36) | 228 (37) | 41 (39) | 0.74 |
| Antiplatelet | 388 (54) | 344 (56) | 44 (42) | 0.011 |
| Anticoagulant | 310 (43) | 239 (39) | 71 (68) | <0.001 |
| **AAD** | 163 (23) | 144 (24) | 20 (19) | 0.38 |
| group1 | 121 (17) | 107 (18) | 14 (13) | 0.33 |
| group3 | 44 (6) | 38 (6) | 6 (6) | 1 |

The abbreviations are as in Table 1.

Variables are presented as mean and SD or median and interquartile range or total numbers and percentages. AAD indicates antiarrhythmic drugs; AF, atrial fibrillation; BMI, body mass index; BNP, B-type natriuretic peptide; BP, blood pressure; CHADS2, congestive heart failure (1 point), hypertension (1 point), age ≥75 years (1 point), diabetes mellitus (1 point), prior stroke or TIA or thromboembolism (2 points); CKD, chronic renal failure (eGFR <60 ml/min/1.73m^2^); COPD, chronic obstructive pulmonary disease; HCM, hypertrophic cardio myopathy; HF, heart failure; LA, left atrial; LVDd, left ventricular end diastolic diameter; LVDs, left ventricular end systolic diameter; LVEF, left ventricular ejection fraction; LVH, left ventricular hypertrophy; MR, mitral regurgitation including moderate MR or severe MR. MRA, mineralocorticoid receptor antagonist; NYHA, New York Heart Association; RAS, renin-angiotensin system; TRPG, tricuspid regurgitation peak gradient

**Supplementary Table 3. Cox proportional hazards regression model for worsening heart failure with age, LA diameter, and LVEF treated as continuous variables.**

|  |  | Multivariable analysis | | | | | | | | | |
| --- | --- | --- | --- | --- | --- | --- | --- | --- | --- | --- | --- |
|  |  | Model 1 | | | | |  | | Model2 | | |
|  |  | HR (95% CI) | | P-value | | |  | | HR (95% CI) | | P-value |
| AF progression |  | 1.64 (1.14 - 2.36) | | 0.008 | | |  | | 1.65 (1.15 - 2.37) | | 0.006 |
| Age |  | 1.22 (1.14 - 1.31) | | <0.001 | | |  | | 1.22 (1.14 - 1.31) | | <0.001 |
| Gender (female) |  | 1.08 (0.85 - 1.37) | | 0.54 | | |  | |  | |  |
| Anemia |  | 1.52 (1.20 - 1.92) | | <0.001 | | |  | | 1.52 (1.21 - 1.92) | | <0.001 |
| Chronic kidney disease |  | 1.21 (0.95 - 1.53) | | 0.12 | | |  | | 1.22 (0.96 - 1.54) | | 0.11 |
| COPD |  | | 0.72 (0.44 - 1.16) | | 0.18 |  | | 0.71 (0.44 - 1.31) | | 0.15 | |
| Previous hospitalization for HF |  | 1.69 (1.34 - 2.11) | | <0.001 | | |  | | 1.70 (1.36 - 2.12) | | <0.001 |
| LA diameter |  | 1.03 (1.01 - 1.05) | | <0.001 | | |  | | 1.03 (1.01 - 1.04) | | <0.001 |
| LVEF |  | 1.00 (0.99 - 1.01) | | 0.73 | | |  | |  | |  |
| Left ventricular hypertrophy |  | 1.27 (1.00 - 1.62) | | 0.05 | | |  | | 1.31 (1.04 - 1.65) | | 0.02 |
| β-blockers |  | 0.77 (0.61 - 0.98) | | 0.03 | | |  | | 0.80 (0.63 - 1.01) | | 0.06 |
| RAS-inhibitors |  | 1.17 (0.92 - 1.47) | | 0.20 | | |  | |  | |  |

A maximum of 16 (2.2%) observations were deleted because of missing variables. AF progression was considered as a time-updated covariate. Age per 5-year increase; LA diameter per 1-mm increase; LVEF per 1% increase. In Model2, covariables were extracted with step wise selection based on AIC. AF, atrial fibrillation; COPD, chronic obstructive pulmonary disease; HF, heart failure; LA, left atrial; LVEF, left ventricular ejection fraction; RAS, renin-angiotensin system.
