# Supplementary material for: Clinical impact of atrial fibrillation progression in patients with heart failure with preserved ejection fraction: A report from the CHART-2 Study
Source: Europace. 2024 Aug 16;26(9):euae218. doi: 10.1093/europace/euae218 (PMC11368130; doi:10.1093/europace/euae218)

Supplementary Figure 1.

Changes in LVEF over time for patients with (A) and without (B) AF progression.


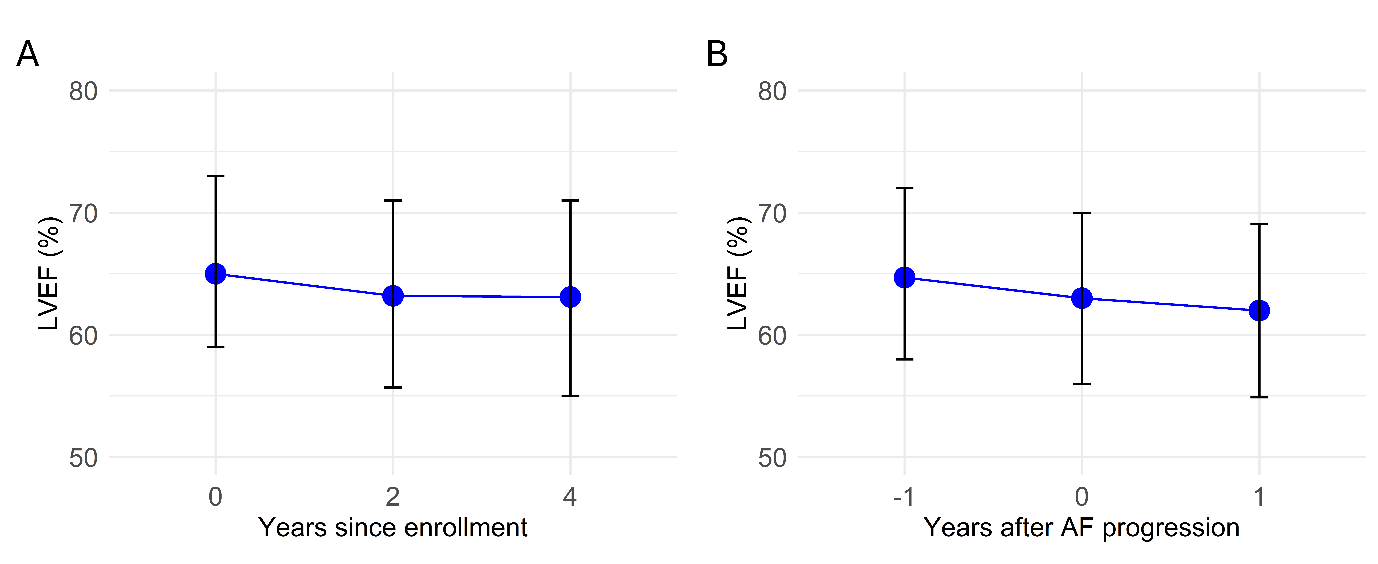


Supplementary Figure 2.

Cox proportional hazards regression model assessing the impact of age on AF progression.

Age was considered as continuous variable.


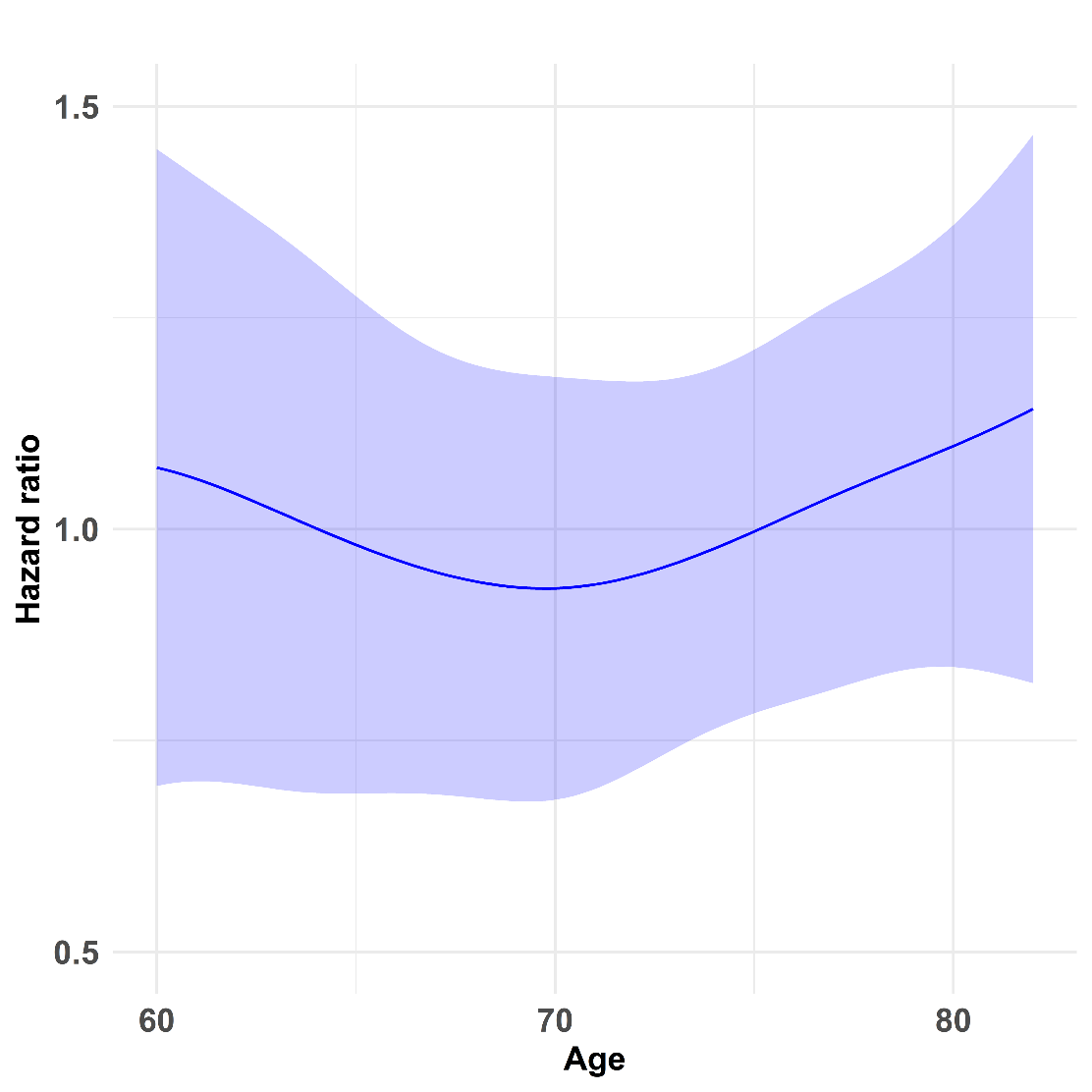

Supplement: euae218_Supplementary_Data [file euae218_supplementary_data.zip › Sullpementary Figure R3.docx]
